# RETRACTION: Effects of Continuing Nursing Intervention on High‐Risk Patients with Diabetic Foot Ulcers: A Meta‐Analysis

**DOI:** 10.1111/iwj.70294

**Published:** 2025-03-03

## Abstract

**RETRACTION:** XuZ. , WuH. , and ShiJ. , “Effects of Continuing Nursing Intervention on High‐Risk Patients with Diabetic Foot Ulcers: A Meta‐Analysis,” International Wound Journal 21, no. 2 (2024): e14683, 10.1111/iwj.14683.PMC1082756642052863

The above article, published online on 30 January 2024, in Wiley Online Library (http://onlinelibrary.wiley.com/), has been retracted by agreement between the journal Editor in Chief, Professor Keith Harding; and John Wiley & Sons Ltd. Following an investigation by the publisher, all parties have concluded that this article was accepted solely on the basis of a compromised peer review process. The editors have therefore decided to retract the article. The authors did not respond to our notice regarding the retraction.

**RETRACTION:** Z. Xu , H. Wu , and J. Shi , “Effects of Continuing Nursing Intervention on High‐Risk Patients with Diabetic Foot Ulcers: A Meta‐Analysis,” International Wound Journal 21, no. 2 (2024): e14683, 10.1111/iwj.14683.PMC1082756642052863

The above article, published online on 30 January 2024, in Wiley Online Library (http://onlinelibrary.wiley.com/), has been retracted by agreement between the journal Editor in Chief, Professor Keith Harding; and John Wiley & Sons Ltd. Following an investigation by the publisher, all parties have concluded that this article was accepted solely on the basis of a compromised peer review process. The editors have therefore decided to retract the article. The authors did not respond to our notice regarding the retraction.

